# Membrane Activity and Channel Formation of the Adenylate Cyclase Toxin (CyaA) of *Bordetella pertussis* in Lipid Bilayer Membranes

**DOI:** 10.3390/toxins12030169

**Published:** 2020-03-10

**Authors:** Oliver Knapp, Roland Benz

**Affiliations:** 1 Department of Life Sciences and Chemistry, Jacobs University, Campus Ring 1, 28759 Bremen, Germany; 2 Rudolf-Virchow-Center, University of Würzburg, Versbacher Str. 9, 97078 Würzburg, Germany

**Keywords:** pore formation, adenylate cyclase toxin, CyaA, *Bordetella pertussis*, membrane interaction, lipid bilayer

## Abstract

The Gram-negative bacterium *Bordetella pertussis* is the cause of whooping cough. One of its pathogenicity factors is the adenylate cyclase toxin (CyaA) secreted by a Type I export system. The 1706 amino acid long CyaA (177 kDa) belongs to the continuously increasing family of repeat in toxin (RTX) toxins because it contains in its C-terminal half a high number of nine-residue tandem repeats. The protein exhibits cytotoxic and hemolytic activities that target primarily myeloid phagocytic cells expressing the αMβ2 integrin receptor (CD11b/CD18). CyaA represents an exception among RTX cytolysins because the first 400 amino acids from its N-terminal end possess a calmodulin-activated adenylate cyclase (AC) activity. The entry of the AC into target cells is not dependent on the receptor-mediated endocytosis pathway and penetrates directly across the cytoplasmic membrane of a variety of epithelial and immune effector cells. The hemolytic activity of CyaA is rather low, which may have to do with its rather low induced permeability change of target cells and its low conductance in lipid bilayer membranes. CyaA forms highly cation-selective channels in lipid bilayers that show a strong dependence on aqueous pH. The pore-forming activity of CyaA but not its single channel conductance is highly dependent on Ca^2+^ concentration with a half saturation constant of about 2 to 4 mM.

## 1. Introduction

**Figure 1:**
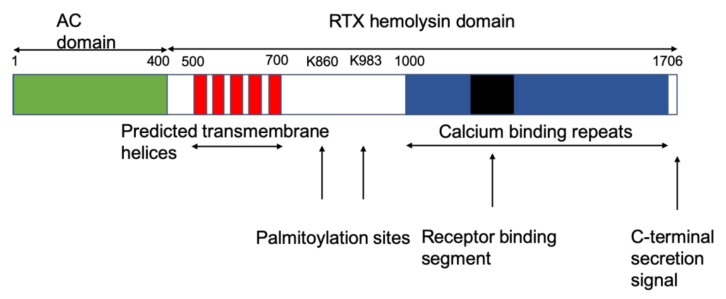
Schematic representation of the different domains of cyclase toxin (CyaA) of *Bordetella pertussis*.

Whooping cough is a highly transmissive disease of the human respiratory tract caused by the Gram-negative bacterium *Bordetella pertussis*. The adenylate cyclase toxin (CyaA) is together with pertussis toxin one of the main secreted virulence factors of this pathogen, besides other factors such as filamentous hemagglutinin, pertactin, fimbria, and tracheal cytotoxin [1]. Adenylate cyclase toxins with minor differences in the calcium-binding domain are also produced by the closely related species *Bordetella parapertussis, Bordetella avium,* and *Bordetella bronchiseptica* [2,3,4]. Differences between the toxins of different *Bordetella* species are mainly in the calcium-binding domain. CyaA is an essential factor in the early stage of bacterial colonization of the respiratory tract [5,6]. It enables the bacteria to escape the host immune system by primarily targeting myeloid lineage cells expressing the α_M_β_2_ integrin receptor (CD11b/CD18) such as macrophages and neutrophils. These cells are the main cellular targets for CyaA [7,8,9,10,11,12]. CyaA differs from other repeat in toxin (RTX) toxins through its assembly and function as the 177 kDa protein exhibits cytotoxic and hemolytic activities (Figure 1). The first 364 N-terminal amino acids constitute an adenylate cyclase (AC) domain, which is translocated through a more or less unknown mechanism across the cytoplasmic membrane into the host cell (Figure 1). Following activation by the intracellular calmodulin (CaM) together with calcium leads to the uncontrolled formation of supraphysiological cyclic adenosine monophosphate (cAMP) levels from ATP, leading to the interruption of several important cellular functions [7,13]. As a result, the microbicidal capacities of the intoxicated cells are debilitated by increased concentration of cAMP followed by cAMP-mediated activations of protein kinase A (PKA) and the exchange factor directly activated by cAMP 1 (EPAC1) [2,12,13]. The AC domain shows some similarities to the adenylate cyclases found in *Bacillus anthracis* (edema factor) and in *Pseudomonas aeruginosa* (ExoY). The sequence similarities and structural isolation of these bacterial adenylate cyclases may hypothesize that the bifunctional CyaA of *B. pertussis* developed by the fusion of an adenylate cyclase and RTX-toxin gene [14]. 

The C-terminal fragment of CyaA with 1332 residues functions as a hemolysin and is able to form small cation selective channels in lipid bilayers (Figure 1) [15,16,17,18]. Thus, the hemolysin part of CyaA contains enough information for cell targeting and pore formation. This part itself is composed of several distinct domains with different functions. The translocation of the AC domain across host plasma membranes is linked to the translocation region, spanning residues 365–500, and is followed by a hydrophobic domain, which is involved in pore formation. It harbors several hydrophobic segments between residues 500 and 700 with potentially amphiphilic and hydrophobic α-helical structures [15,16,17,18,19,20,21,22]. This region accounts for membrane insertion and shows some similarities to the pore-forming region of other RTX-toxins, e.g., the *Escherichia coli* α-hemolysin HlyA [5,23]. 

The acylation region spans from residue 750 to 1000 and contains the two main acylation sites at lysines K860 and K983 [24,25,26,27,28,29]. Hackett et al. [24] showed that CyaA is post-translationally modified by palmitoylation on Lys 983 (Figure 1). Later on, it was found that the recombinant CyaA toxin produced in *E. coli* K-12 was also palmitoylated at K860 in the presence of the CyaC protein, which is an acyl-translational enzyme encoded by *cyaC* of the adenylate cyclase operon [25,26,27,28,29]. Through the enzyme action of CyaC, palmitoyl residues are covalently attached to the ε-amino group of one or both of the two lysine residues at positions 860 and 983, which correspond to highly conserved residues among RTX toxins [25,28,29]. Acylation is essential for tight binding and interaction with its target cell receptors as well as for other toxin activities such as the modulation of toxin oligomerization [30,31]. However, acylation does not seem to be highly important for pore formation, as the nonacylated CyaA precursor toxin also forms pores—with a reduced frequency—in planar lipid bilayers and naked liposome membranes with similar properties as the pores formed by acylated toxin [16,25]. 

The next domain between residues 913 and 1612 contains about 45 glycine and aspartate-rich nonapeptide tandem repeats of the form of the type G-G-X-G-(N/D)-D-X-(L/I/F)-X (where X represents any amino acid) that form calcium-binding sites. These repeats are typical for RTX toxins (see Figure 1) and are involved in receptor binding [32,33,34,35,36,37]. The region comprised by residues 1166–1281 within this glycine-rich repeat is required for binding to the αMβ2 integrin receptor [38,39,40] (Figure 1). 

Each repeat binds a single calcium ion with a binding constant between 0.5 and 0.8 mM [41]. CyaA has about 45 of these binding sites of low affinity besides a few high-affinity binding sites, which could not be localized in CyaA [41]. Circular dichroism spectroscopy analysis revealed that calcium binding is associated with a conformational change of CyaA involving an important increase in the content of alpha-helical structures [42]. Other studies showed that calcium binding induces the formation of parallel β-roll motifs, where β-strands of two parallel sheets are connected by calcium binding. This results in the formation of a right-handed spiral within the C-terminal domain, which seems to be necessary for cell intoxication [33,34,41,42]. 

The last about 50 to 60 amino acids from the C-terminal end of CyaA (see Figure 1) represent the export signal because it is actively secreted from *B. pertussis* by a specific type I transport system [43,44]. It consists of the products of the genes *cyaB* and *cyaD*, which are homologues of the proteins involved in *E. coli* hemolysin export machinery [43,44,45]. These genes are part of the operon *cyaCABDE* (in transcriptional order) [43], which contains also the gene *cyaE* of an outer membrane protein with a function similar to the outer membrane protein TolC of *E. coli* [46,47]. The activated soluble protein—the main part remains bound to the outer membrane of the bacteria and stays inactive [48]—interacts then with the surface of the target cells. Recent results showed that the RTX toxins CyaA, HlyA, and LtxA exhibit a weak lectin activity and interact with the N-linked oligosaccharides of their β2 integrin receptors [40]. This raises the possibility that the initial unsaturable binding of RTX cytotoxins to various cells might occur through the recognition of glycosylated membrane components such as glycoproteins and gangliosides.

## 2. Membrane Interaction of CyaA: Membrane Binding, Adenylate Cyclase (AC) Translocation, and Pore Formation

The interaction of CyaA with target cell membranes starts with the reversible adsorption of the toxin by the membrane via electrostatic interactions [18,49] followed by an irreversible membrane insertion. Once CyaA has inserted into the cell membrane, it suffers an irreversible conformational change after which it cannot be recovered from the membrane without the use of detergents [41]. Studies with the isolated calcium-binding domain of the HlyA toxin of *E. coli* revealed that this part of the protein might adsorb on the membrane in the early stages of HlyA–membrane interaction [50].

After membrane binding and insertion, the AC penetrates into the host cell cytosol were the N-terminal AC domain of CyaA binds intracellular calmodulin and starts with the calmodulin- dependent cAMP production [7,13]. Through this specific enzymatic activity, cAMP is increased approximately 10,000-fold [7,51] and the subsequent bactericidal functions of phagocytes, e.g., phagocytosis and a decrease of chemotactic and oxidative burst capacities, are significantly blocked [52,53,54]. Moreover, the toxin can induce apoptosis in macrophages [10,11] by a mechanism that involves the disruption of the mitochondrial membrane potential [55]. Veneziano et al. demonstrated that CyaA does not require any specific eukaryotic components apart from calmodulin to translocate its catalytic domain across a membrane [49]. The crystal structure of the AC domain of CyaA was recently solved in complex with the C-terminal fragment of calmodulin [56].

Unlike most other enzymatically active toxins, the entry of this part of the molecule is not dependent on the receptor-mediated endocytosis, and it has been shown in several different studies that the adenylate cyclase penetrates the target cell directly across the cell membrane of a variety of different cell types [57,58,59,60,61]. Fiser et al. showed that this unique translocation of the AC domain of CyaA also causes a novel type of membrane path for Ca^2+^ influx into monocytic cells [62]. This mechanism is independent of the pore formation and enzymatic activity of CyaA or the release of Ca^2+^ from intracellular stores [62]. The unique calcium influx pathway induces the cleavage of talin by calpain and enables the mobilization of the CyaA-CD11b/CD18 complex into lipid rafts. Lipid raft environments enriched in cholesterol appear to support the translocation of the enzymatic domain across the host cell membrane [63]. Veneziano et al. demonstrated that CyaA can be translocated across tethered lipid bilayers [49]. The translocation of the catalytic domain seems to be driven in part by the electrical field across the membrane in a calcium-dependent manner [49]. The translocation region is hereby of crucial interest, since arginine residues from the segment 454–484 within this region are involved in membrane interaction, folding, and the permeabilization of CyaA and its enzymatic domain [64]. Voegele et al. propose that this region induces a local destabilization of the membrane, thereby decreasing the energy required to translocate the catalytic domain across the plasma membrane [64].

Several groups have shown that antigens “inserted and hidden” within the amino acid sequence of the AC domain can be successfully delivered to the major histocompatibility complex-I (MHC I) complex [38,65]. Therefore, interest in CyaA increased significantly, and it is thought that it could be useful for the development of new vaccinations against different viruses or cancer. Another enzymatic activity was recently assigned to CyaA by González-Bullón et al., who reported that CyaA exhibits a phospholipase A activity that could destabilize the membrane to facilitate the membrane translocation of the AC domain [66]. However, others could not verify this claim. Bumba et al. and Voegele et al. could not detect any phospholipase A activity associated with the CyaA polypeptide [67,68]. It was shown that the two putative conserved phospholipase A catalytic residues Ser606 and Asp1079 are not involved in the process of membrane translocation of the AC domain of CyaA across target membranes [67]. However, González-Bullón et al. reproduced and corroborated their first observations of the CyaA–PLA activity and of the involvement of Ser606 as a catalytic site for CyaA PLA activity and its involvement in AC translocation [69].

**Figure 2:**
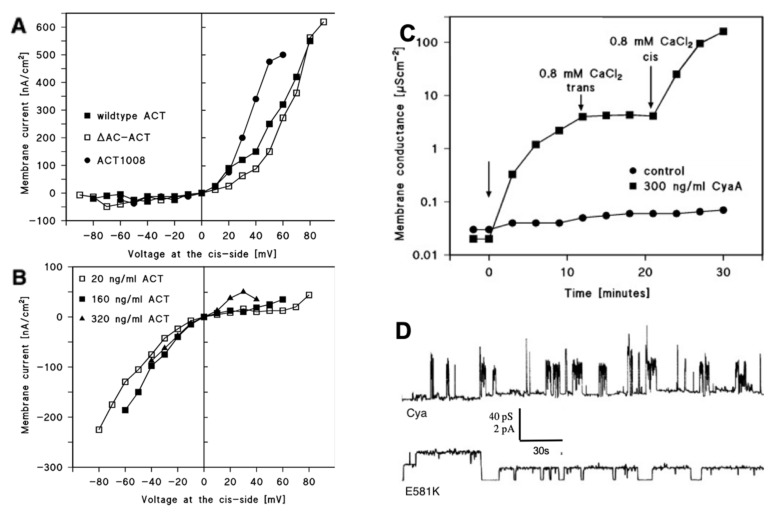
(**a**) Current–voltage relationships of cyclase toxin (CyaA) pores initiated by positive potential (+ 50 mV) applied to the cis side. The membranes were formed from asolectin/n-decane. The aqueous phase contained 150 mM KCl and 10 mM 2-(4-(2-Hydroxyethyl)-1-piperazinyl)-ethanesulfonic acid-potassium hydroxide (HEPES-KOH) pH 7; the temperature was 20 °C. The cis side contained in addition 320 ng/mL wild-type CyaA (full squares), 320 ng/mL ΔAC-CyaA (open squares), or 320 ng/mL CyaA 1008 (full circles) (adapted from [78]). (**b**) Current–voltage relationships of CyaA pores initiated by negative potential (−50 mV) applied to the cis side. The membranes were formed from asolectin/n-decane. The aqueous phase contained 150 mM KCl and 10 mM HEPES-KOH pH 7; the temperature was 20 °C. The cis side contained either 20 ng/mL wild-type CyaA (full circles), 160 ng/mL wild-type CyaA (full squares), or 320 ng/mL wild-type CyaA (full triangles) (adapted from [78]). (**c**) Increase of membrane conductance after the addition of 300 ng/mL CyaA to the *cis* side of a black asolectin/n-decane membrane (left side arrow) and of 0.8 mM Ca^2+^ first to the *trans* side of the membrane (middle arrow) and then to the *cis* side of the membrane (right side arrow). The aqueous phase contained 1 M KCl, pH 7 on both sides of the membrane. The addition of 300 ng/mL and CyaA added to the *cis* side of the membrane (left-hand side arrow) resulted in an increase of the membrane conductance (full squares) by a factor of about 100 over control in the absence of CyaA (full points). The addition of 0.8 mM CaCl_2_ to the *trans* side (middle arrow) had no impact on membrane conductance, whereas the addition of 0.8 mM CaCl_2_ to the *cis* side (right-hand side arrow) led to a dramatic increase of membrane conductance by many orders of magnitude (full squares). The temperature was 20 °C and 50 mV were applied to the *cis* side (adapted from [18]). (**d**) Single-pore recordings of asolectin/n-decane membranes in the presence of a 13 ng/mL concentration of the purified CyaA-E581K protein (lower line) or intact recombinant CyaA (upper line). Mutant E581K showed an increased pore lifetime, which seems to be linked to an increased hemolytic activity. The applied membrane potential was 50 mV, and the temperature was 20 °C (adapted from [72]).

Furthermore, the hydrophobic domain of the N-terminal part of CyaA is able to form small cation-selective transmembrane channels of a defined size and a short pore lifetime of a few seconds (Figure 2d) [16,17,69,70,71], and it also causes osmotic cell lysis [15]. Dose–response analyses indicated that the lytic activity on target cells is a highly cooperative function of toxin concentration (Hill number ≥ 3) and slow kinetics (lag time of >1 h), suggesting that oligomerization was involved in RTX toxin pore formation [17,71,72,73,74,75]. Moreover, in vitro complementation experiments with pairs of individually inactive deletion variants allowed restoring, at least in part, the hemolytic and cytotoxic activities, suggesting that two or more toxin molecules associate to form a pore [76,77]. However, Gray et al. observed that CyaA induced a rapid increase in K^+^-efflux from sheep erythrocytes and Jurkat cells [74]. The dependence of this process on toxin concentration together with the analysis of the time course suggested that CyaA monomers are sufficient to induce such a K^+^-efflux. The author agreed that pore formation requires oligomerization and state that the AC translocation-related K^+^-efflux is separate and distinct from the structure required for intoxication [74].

Pore formation of CyaA was extensively studied with the help of hemolysis assays with osmotic protectants and conductance measurements in black lipid membranes. It was concluded from these studies that CyaA forms transient small cation-selective pores with a diameter of only 0.6–0.8 nm in contrast to other RTX toxins, such as HlyA of *E. coli* that has a pore diameter of 2.0–3.0 nm [16,17,79,80]. These results are in line with its relatively low hemolytic activity [81].

**Figure 3:**
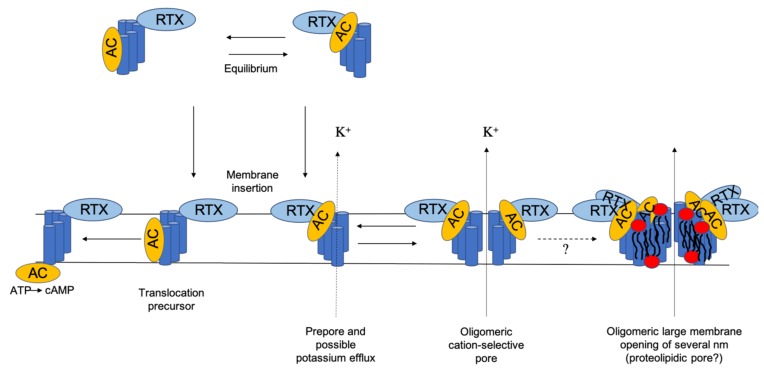
Schematic model of CyaA action on target membranes. Adapted from Osickova et al. [71].

However, the exact mechanism of AC delivery and hemolysis/pore formation is still not completely understood, and for a long time, there was some disagreement over whether the translocation of the enzymatic component and the oligomerization into cation-selective channels are two distinct mechanisms or if both processes are needed for a successful intoxication. Nowadays, it is assumed that translocation of the enzymatic domain and channel formation are two independent processes, as Rogel and Hanski [13] showed that both activities compete with each other and that the translocation of the AC domain can be uncoupled from membrane insertion of the toxin at low temperature. The full activity can be restored by raising the temperature or adding free calcium [13]. The internalization of AC seems to be a monomolecular process, while CyaA channel formation involves more than one CyaA molecule in vivo and in vitro [16,17,32,71,82,83]. A model established by Osickova et al. suggests that the water-soluble toxin exists in two conformational isomers [71]. After membrane insertion, one isomer mediates the translocation of the catalytic domain, whereas the other one represents a channel precursor (see Figure 3) [71]. This means that AC translocation and the pore-forming activities of CyaA are supposed to be independent and occur in parallel in target cell membranes [71]. One CyaA precursor translocates the AC enzyme domain into the target cell cytosol across the plasma membrane and catalyzes the conversion of cytosolic ATP to cAMP. The other precursor forms a prepore, which might be already able to induce a potassium efflux [74]. The precursor pore finally oligomerizes into a fully functional cation-selective CyaA pore that permeabilizes the membrane bilayer for the efflux of cytosolic potassium ions. The two conformers might exist in an equilibrium, which can be influenced by different factors such as temperature, acylation status, free calcium concentration, antibody binding, and substitution mutations [13,41,42,71,72,74]. According to González-Bullón et al. and Ostolaza et al., these pores increase over time in dependence of toxin concentration and can evolve into large membrane openings of several nm wide possibly with the participation of membrane lipids (proteolipidic pores) [84,85].

According to this model, hemolysis occurs after membrane adsorption, insertion, and oligomerization of the channel precursor proteins [71,79]. Segments with amphipathic and/or hydrophobic helix-like structures (I: 502–522, II: 529–550, III: 571–593, IV: 607–627, V: 678–698), which possibly insert into host cell membranes, have been predicted for the secondary structure of CyaA [20,71]. Deletions within this region (residue 623–780 and 827–887) prevent the translocation of the AC into the host cell and reduce the hemolytic activity of CyaA [81]. Similarly, it has been shown that tyrosine 940 plays an important role for the CyaA–membrane interaction [86]. The lack of the AC domain (1–373) or the C-terminal nonapeptide-rich part (1009–1706) has no influence on the channel properties [86]. The point mutations introduced in these predicted transmembrane structures were found to affect the ability to form pores and play a critical role in cell binding, formation of cation-selective pores, and the translocation of the enzymatic subunit (Figure 2d) [62,71,75]. Mutations affecting the glutamates at position 509 or 516, which are located in a predicted α-helical transmembrane structure in helix I, show a significant altering of the channel properties and the protein translocation [71]. Whereas neutral substitutions have only little effect on the toxin activities, charge exchange by lysine (E509K and E516K) reduces the translocation rate of the catalytic domain as well as the hemolytic activity, ion selectivity, and channel-forming capacity. The substitution of E509 by a helix-breaking proline abolishes totally the invasion of the AC domain, whereas channel formation and cell binding remain unaffected in red blood cells. This is a strong indication that this segment is involved in AC delivery as well as pore formation [71]. The double mutation E509K/E516K further enhanced the hemolytic and pore-forming activity of CyaA [71]. Another pair of glutamate residues, E570 and E581 in helix III, has also been shown to affect CyaA pore-forming activity. Mutant E581K enhanced the hemolytic and pore-forming activity by increasing both the frequency of formation and lifetime of toxin pores (Figure 2d), and double mutation E570K/E581P reduced the specific hemolytic activity [75]. A negative charge at position 570, but not at position 581, was found to be essential for the cation selectivity of the pore, suggesting that E570 might act as ion filter inside or close to the pore mouth [73]. Kurehong et al. focused on the same hydrophobic region and could confirm these results. Mutation of the polar amino acids Q574 and E581 has an influence on the pore-forming activity of CyaA [87]. Similarly, a considerable influence in the membrane penetration and pore-forming activity of CyaA was found for residues 529 to 549 in segment II [19].

Similar results were obtained in studies using the monoclonal antibody 3D1, which recognizes an epitope (amino acids 373–399) at the distal end of the CyaA catalytic domain. The translocation of AC to the cytosol of erythrocytes was inhibited through the binding of the antibody, whereas hemolytic activity increased three to fourfold [88]. A “hyperhemolytic” phenotype was also achieved by the deletion mutant CyaA∆N489 in which the catalytic domain, along with additional amino acids distal to it, was eliminated. This might lead to the assumption that preventing the AC translocation favors a toxin conformation that is more suitable for pore formation [88]. Masin et al. could also achieve a hyperhemolytic CyaA after the substitution of negatively charged residues located in the linker segment of CyaA (residues 400 to 500 of CyaA), which forms an α-helical structure interacting with the lipid bilayer [88]. The authors suggest that this “AC to Hly-linking segment” may be responsible for the much smaller conductance and permeability of CyaA pores, as compared to typical RTX hemolysins [89].

Substitution mutations in the putative helix II affect the hemolytic capacity of CyaA without affecting the AC translocating activity (G531P), whereas the substitution of G537, A538, or A546 by diverse residues selectively impaired the ability to translocate the AC domain across the cell membrane [19]. These substitution mutants were still capable of forming transmembrane pores, and therefore, the hemolytic capacity was not affected. The replacement of A538 by proline eliminated the voltage-activated increase of membrane conductivity of CyaA in asolectin membranes [87]. These results show that the linker segment may interact with the structure of helix II and control the formation of CyaA pores [87,88]. Another study proposed that three glycine residues in helix II (G530, G533, and G537) might be a crucial component of the CyaA pore structure and have a role in CyaA oligomerization [90].

Immunolabeling in combination with blue-native polyacrylamide gel electrophoresis (BN-PAGE) of erythrocytes membranes treated with CyaA revealed the presence of rather unstable CyaA oligomers in the erythrocyte membrane with apparent molecular masses of 200, 300, 410, and 470 kDa [75]. Some of them (410 and 470 kDa) were attributed to the oligomers of the 200 kDa CyaA toxin [75]. This study also revealed a correlation between the oligomerization of CyaA mutants in the membrane, their pore-forming capacity, and their specific hemolytic activity. Mutants of CyaA that showed an enhanced (E581K) or reduced (E570K/ E581P) pore-forming activity and pore lifetime also possessed an enhanced or reduced ability to form oligomers in the cell membranes of erythrocyte [73]. New results question this prevailing static model of cell membrane permeabilization by CyaA and propose a new complex and dynamic model in which membrane permeabilization depends on a number of factors, including membrane lipid composition, temperature, time, and toxin concentration (Figure 3) [84,91,92]. González-Bullón et al. showed in cell-sized membrane model systems that CyaA can form—as an initial step—small membrane lesions that can increase in size over time, depending from the available amount of toxin. Apparently, these small lesions can finally develop large membrane pores of several nm in size. These membrane ruptures might then become eventually large enough to allow the influx of molecules with large molecular mass such as fluorescent dextrans. During this whole process, the vesicle integrity seems to be preserved [84]. This goes in line with a study by Masin et al., where a large-scale membrane disruption by CyaA and the leakage of fluorescein isothiocyanate (FITC) from large, unilamellar vesicles (LUVs) was proposed [91]. González-Bullón et al. reported also that they were able to resolve protein bands with apparent molecular masses of ≈550, 800, 1000, and 1200 kDa by BN–PAGE, both in lipid vesicles and in cells (macrophages and CR3-negative cells) under lytic conditions [84]. The observed protein bands were interpreted as CyaA trimers, tetramers, pentamers, and hexamers [84], and these are in agreement with studies showing that the hemolysis of CyaA is a cooperative event with a hill number ≥ 3 [17]. However, the reported BN–PAGE bands are somewhat bigger then the previously ones reported by Vojtova-Vodolanova et al. [75]. The reason for this discrepancy is explained by the shorter exposition time of CyaA to sheep erythrocytes (30 min), and it is assumed that a longer exposer time would have resulted in larger CyaA oligomers [84]. It was speculated by Ostolaza et al. in a recent review on biological activity of RTX toxins [85] and González-Bullón et al. [85] that CyaA pores can be as large as listeriolysins and toroidal (proteolipidic) pores. Both studies postulate a “sequential” mode of oligomerization, which involves the successive addition of units with the same stoichiometry (Figure 3) [84,85].

The same group through atomic force microscope (AFM) imaging could confirm these results. Different CyaA structures of heterogeneous composition were detected. They formed lines, arcs, or closed rings in phosphatidylcholine vesicles. These structures are believed to be transmembrane pores with variable diameters of up to ≈ 20 nm in the narrowest and of up to ≈ 50–60 nm in the more external part [84]. So far, any other groups have not repeated these results. It remains to be elucidated why the hemolytic activity on red blood cells is small when such large transmembrane structures are formed. However, it appears that the invasive AC activity and pore formation synergize and increase the potency of the overall toxicity of CyaA on target cells, in particular on CD11b+ cells [61,93]. This has probably to do with the influx of Ca^2+^ ions through the CyaA channels and the subsequently increased activity of the AC domain to form cAMP.

## 3. Factors Affecting CyaA Channel-Forming Activity: Effect of Calcium Ions, Membrane Potential, and pH

Free calcium ions at concentrations above 0.1 mM Ca^2 +^ induce important conformational changes of the repeat domain, which are essential for the CyaA biological activity [33,34]. This includes target cell binding, the hemolytic activity, and the capacity to translocate the enzymatic domain of AC of *B. pertussis* into host cells [41,94]. Rogel and Hanski showed that after a single exposure to calcium, CyaA becomes competent for membrane insertion and hemolytic activity, even without free calcium ions, assuming that calcium ions are firmly bound to CyaA [13].

Black lipid bilayer experiments with membranes made from asolectin proved that the conductance of CyaA channels is independent of the calcium concentration within the range between 0.5 and 10 mM CaCl_2_ (about 40 pS in the presence or absence of CaCl_2_) [18]. When CaCl_2_ concentration was raised to 0.8 mM on the cis-side—the side of the addition of CyaA—the membrane conductance increased substantially by more than 1000–fold (Figure 2c) [18]. This suggests that calcium ions influenced only the frequency of channel formation but not its conductance [18]. The minimum effective calcium concentration necessary to mediate this increase was about 0.6–0.8 mM CaCl_2_, depending on the ionic strength of the aqueous phase. The maximum calcium ion-mediated effect on the increase of membrane conductance was reached at about 10 mM CaCl_2_ with a half saturation constant of about 2 to 4 mM. When the repeat domain of CyaA was partially or fully deleted, the addition of CaCl_2_ did not influence the frequency of channel formation [18]. Additional experiments on target cells or black lipid bilayer membranes with fragments derived from the RTX domain were able to show that these fragments complement inactive CyaA molecules that lack different blocks within the repeat domains [95]. Full biological activity was achieved for the truncated CyaA variant CyaA (1–1490) when complemented with the two partial RTX-domain derived sequences CyaA (1006–1706) (full length RTX-sequence) or CyaA (1490–1681) being added to the experimental setup in the presence of 1 mM CaCl_2_ [95]. The addition of the partial RTX sequences had no influence on membrane activity of the truncated form when calcium ions were absent in the experiments. These results were supported by studies where truncated forms of CyaA were not able to intoxicate target cells or where an inactive truncated CyaA molecule that lacks the 76 C-terminal amino acids could be fully restored to its toxic activity when a polypeptide comprising these 217 C-terminal residues of CyaA was added to the inactive CyaA derivative [76,77]. However, lipid bilayer experiments with CyaA in the presence of calmodulin suggested that it was not able to interfere with the calcium-dependent activation of CyaA pores [78].

The membrane potential is another important factor for the biological activity of CyaA. Patch clamp studies showed that the AC delivery into certain target cells was dependent on a negative membrane potential [96]. Atrial cells could only be successfully intoxicated when held at a negative potential, whereas under positive potential, no toxin activity could be observed [96]. Knapp et al. could show that CyaA forms different pores in lipid bilayer experiments depending on the orientation of the electrical potential across the membrane [78]. When CyaA was added at the membrane side facing a positive potential, pores with a defined size were formed and conductance increased rather rapidly. However, when the toxin was added to the side at negative potential, CyaA behaved more detergent-like and a showed reduced ability to form pores [78]. Experiments with the deletion mutation of CyaA (ΔAC and ACT 1008, i.e., deletions of amino acids 1008 to 1490) proved that the voltage sensor must be located within the residues 400 and 1008 (most likely in the pore-forming segment), since these deletion mutants behaved similar to wild-type CyaA (Figure 2a). Pore formation under positive potential resulted in a strongly increasing membrane current, whereas when pore formation occurred under negative potential, the membrane current decreased or remained at a constant level (Figure 2a,b) [78]. Moreover, calcium ions in millimolar concentration were found to enhance the voltage dependence of CyaA pores [78]. Veneziano et al. observed similar results when studying the translocation of the AC domain across tethered lipid bilayers; the translocation of the AC domain was strictly dependent on the presence of calcium and the application of a negative electrical potential across the membrane [49]. However, CyaA provokes colloid-osmotic cell lysis or delivers its AC domain efficiently even into cells with low membrane potential, such as sheep erythrocytes [7,97]. As discussed above, it is also able to release or allow an influx of marker substances from multilamellar liposomes devoid of membrane potential [84,91,98]. Whether the lack of a membrane potential is responsible for the observed CyaA structures in membrane, as postulated by González-Bullón et al. [84] or not, remains to be open at present and needs to be addressed in further studies.

The single-pore conductance was strongly affected by the variation of pH and increased in 1 M KCl with increasing pH from about 4 pS at pH 5 to about 60 pS at pH 9 [78]. The ion selectivity remained unaffected by pH. Experiments with CyaA mutants revealed that the adenylate cyclase (AC) and repeat (RT) domains were not involved in voltage and pH sensing [78].

## 4. Conclusions

The adenylate cyclase toxin of *B. pertussis* is one of the main virulence factors of this bacterium and was studied intensively in the last three decades. However, the exact mechanism of CyaA transport into the target cells, which is also interesting for biotechnological applications [65], is still not fully understood. The current model of the activity of CyaA—membrane binding via receptor or not, membrane insertion, translocation of the AC domain and formation of an oligomeric pore—is challenged by results regarding the new enzymatic activities of CyaA and the mechanisms of membrane permeabilization, of pore assembly, and its relation to the translocation of the AC domain. In order to reconcile these new results with the older ones, it is important that these results will be verified by other groups and by the use of different methods. A special focus has to be set on pore formation, since it is possible that the pore-forming activities of RTX toxins allow the permeation of small molecules through the target cell membrane, including calcium ions, which start the inflammatory reactions and possibly cell lysis [99,100,101]. The activity and the biophysical characteristics of pore formation by CyaA may trigger a strong impact of the toxin’s bacterial virulence. Nevertheless, it has to be considered that the pore-forming activity of RTX-toxins, although being essential for virulence, may represent an indirect mechanism of toxicity in the case of CyaA. Unfortunately, not too much is known about the 3D structure of the RTX toxin channels. For this reason, it must be speculated for an individual RTX toxin whether a single molecule or an oligomer is involved in transmembrane pore formation. However, at least for the hemolysin of *E. coli* and CyaA of *B. pertussis*, it has been shown that the pore-forming unit is an oligomer [75,102].
